# Insertion of an SVA-E retrotransposon into the *CASP8* gene is associated with protection against prostate cancer

**DOI:** 10.1093/hmg/ddv622

**Published:** 2016-01-05

**Authors:** Simon N. Stacey, Birte Kehr, Julius Gudmundsson, Florian Zink, Aslaug Jonasdottir, Sigurjon A. Gudjonsson, Asgeir Sigurdsson, Bjarni V. Halldorsson, Bjarni A. Agnarsson, Kristrun R. Benediktsdottir, Katja K.H. Aben, Sita H. Vermeulen, Ruben G. Cremers, Angeles Panadero, Brian T. Helfand, Phillip R. Cooper, Jenny L. Donovan, Freddie C. Hamdy, Viorel Jinga, Ichiro Okamoto, Jon G. Jonasson, Laufey Tryggvadottir, Hrefna Johannsdottir, Anna M. Kristinsdottir, Gisli Masson, Olafur T. Magnusson, Paul D. Iordache, Agnar Helgason, Hannes Helgason, Patrick Sulem, Daniel F. Gudbjartsson, Augustine Kong, Eirikur Jonsson, Rosa B. Barkardottir, Gudmundur V. Einarsson, Thorunn Rafnar, Unnur Thorsteinsdottir, Ioan N. Mates, David E. Neal, William J. Catalona, José I. Mayordomo, Lambertus A. Kiemeney, Gudmar Thorleifsson, Kari Stefansson

**Affiliations:** 1 deCODE genetics/AMGEN, Sturlugata 8, 101 Reykjavik, Iceland,; 2 Institute of Biomedical and Neural Engineering, School of Science and Engineering, Reykjavik University, 101 Reykjavik, Iceland,; 3 Landspitali-University Hospital, IS-101 Reykjavik, Iceland,; 4 Faculty of Medicine,; 5 Department of Anthropology and,; 6 School of Engineering and Natural Sciences, University of Iceland, IS-101 Reykjavik, Iceland,; 7 Netherlands Comprehensive Cancer Organisation, 3501GD Utrecht, The Netherlands,; 8 Radboud University Medical Center, Radboud Institute for Health Sciences, 6500HBNijmegen, The Netherlands,; 9 Division of Medical Oncology, Ciudad de Coria Hospital, 10800 Coria, Spain,; 10 Division of Urology, NorthShore University Health System, Evanston, IL 60201, USA,; 11 Department of Urology, Northwestern University Feinberg School of Medicine, Chicago, IL 60611, USA,; 12 School of Social and Community Medicine, University of Bristol, Bristol BS8 1TH, UK,; 13 Nuffield Department of Surgical Science, University of Oxford, John Radcliffe Hospital, Oxford OX3 9DU, UK,; 14 University of Medicine and Pharmacy Carol Davila, Theodore Burghele Urology Clinic, Str. Dionisie Lupu, No.37, 020021Bucharest, Romania,; 15 Department of Dermatology, Medical University of Vienna, Währinger Gürtel 18-20, A-1090Vienna, Austria,; 16 Icelandic Cancer Registry, Skogarhlid 8, 105 Reykjavik, Iceland,; 17 University of Medicine and Pharmacy Carol Davila, St Mary General Surgical Clinic, Blv. I. Mihalache 29-43, 011172Bucharest, Romania,; 18 Oncology Centre, Addenbrooke's Hospital, University of Cambridge, Cambridge CB2 0QQ, UK and; 19 Division of Medical Oncology, University of Colorado, Aurora, CO 80045, USA

## Abstract

Transcriptional and splicing anomalies have been observed in intron 8 of the *CASP8* gene (encoding procaspase-8) in association with cutaneous basal-cell carcinoma (BCC) and linked to a germline SNP rs700635. Here, we show that the rs700635[C] allele, which is associated with increased risk of BCC and breast cancer, is protective against prostate cancer [odds ratio (OR) = 0.91, *P* = 1.0 × 10^−6^]. rs700635[C] is also associated with failures to correctly splice out *CASP8* intron 8 in breast and prostate tumours and in corresponding normal tissues. Investigation of rs700635[C] carriers revealed that they have a human-specific short interspersed element-variable number of tandem repeat-*Alu* (SINE-VNTR-*Alu*), subfamily-E retrotransposon (SVA-E) inserted into *CASP8* intron 8. The SVA-E shows evidence of prior activity, because it has transduced some *CASP8* sequences during subsequent retrotransposition events. Whole-genome sequence (WGS) data were used to tag the SVA-E with a surrogate SNP rs1035142[T] (*r*^2^ = 0.999), which showed associations with both the splicing anomalies (*P* = 6.5 × 10^−32^) and with protection against prostate cancer (OR = 0.91, *P* = 3.8 × 10^−7^).

## Introduction

*CASP8* encodes an initiator caspase that functions in the extrinsic pathway of apoptosis, controlled by death receptors of the tumour necrosis factor receptor superfamily. Somatic deletions and point mutations of *CASP8* have been observed in several cancer types including colorectal, head and neck, gastric cancer and neuroblastoma (1,2). Germline sequence variants in and near the *CASP8* gene have been implicated in susceptibility to several different cancer types including breast cancer, melanoma, basal-cell carcinoma (BCC) and chronic lymphocytic leukaemia (3–7).

We have sequenced the genomes of 8383 Icelanders and imputed the genotypes of 150 656 chip-typed, long-range phased Icelanders and their relatives. This allows us to search for associations between over 25 million sequence variants and a broad range of phenotypes (7,8). Recently, we reported an association between variants near *CASP8* and BCC. One of the BCC risk variants, rs700635[C], is strongly associated with transcriptional and splicing anomalies involving *CASP8* (7). Here, we show that rs700635[C] tags an SVA-E retrotransposon that is inserted into intron 8 of the *CASP8* gene and is linked to the observed splicing anomalies. Surprisingly, we find that insertion of the SVA-E is also associated with protection against prostate cancer.

## Results

We previously found that the BCC risk variant rs700635[C] (Icelandic population allele frequency: 0.299) is associated with impaired splicing of *CASP8* intron 8. A highly correlated variant (*r*^2^ = 1 in Iceland), rs1830298 has been shown to be associated with breast cancer (9) and we confirmed this effect for rs700635[C] in 5534 breast cancer patients and 309 172 controls from Iceland [odds ratio (OR) = 1.08, *P* = 0.0035, 95% confidence interval (CI): 1.02–1.13]. Given the potential for multi-cancer susceptibility conferred by this variant, we tested its association with other forms of cancer, using Icelandic Cancer Registry (ICR) records. We saw a signal suggesting that rs700635 is associated with prostate cancer (Table 1) based on 5274 cases and 97 905 controls. However the OR indicated that, unlike for BCC and breast cancer, the rs 700635[C] allele appeared to protect against prostate cancer (OR = 0.92, *P* = 0.0018, 95% CI: 0.87–0.97). We sought to confirm this by examining prostate cancer genome-wide association study chip data from the Netherlands and by single-track genotyping rs700635 in replication samples from the USA, Spain, Romania and the UK, a total of 5245 non-Icelandic prostate cancer cases and 9971 controls (Table 1). The replication sample set corresponds to an effective sample size of 6441 cases and 6441 controls, providing an estimated power of 87% to reproduce the association. The initial finding was confirmed in the replication sample set (OR = 0.90, *P* = 1.4 × 10^−4^, 95% CI: 0.85–0.94). Overall evidence for the association, including the Icelandic sample, was OR = 0.91, *P* = 1.0 × 10^−6^, 95% CI: 0.88–0.95 and was without significant heterogeneity (Table 1). The published (9) breast cancer risk allele, rs1830298[C] showed similar protection against prostate cancer in Iceland (OR = 0.92, *P* = 0.0018) as would be expected from the strong correlation between the two variants.

**Table 1.** The association of rs700635 and rs1035142 with prostate cancer

Sample Set	Number of cases	Number of controls	rs700635[C]					rs1035142[T]				
			Frequency in controls	OR	95%CI	*P*	*P*_het_^a^	Frequency in controls	OR	95%CI	*P*	*P_het_*^a^
Iceland^b^	5274	97 905	0.299	0.92	(0.87–0.97)	0.0018		0.379	0.93	(0.89–0.98)	0.0064	
Netherlands	1445	3746	0.288	0.84	(0.76–0.92)	3.0 × 10^−4^		0.399	0.83	(0.76–0.91)	3.6 × 10^−5^	
Spain	729	1923	0.288	0.85	(0.74–0.98)	0.021		0.413	0.94	(0.83–1.06)	0.32	
UK	543	1889	0.263	0.84	(0.72–0.98)	0.030		0.364	0.81	(0.70–0.93)	0.0038	
USA	1547	1291	0.277	1.03	(0.92–1.16)	0.610		0.393	0.97	(0.87–1.08)	0.54	
Romania	981	1122	0.286	0.95	(0.83–1.09)	0.480		0.442	0.94	(0.83–1.06)	0.30	
**All non-Icelandic**^c^	**5245**	**9971**		**0.90**	**(0.85–0.94)**	**1.4 × 10^−4^**	**0.053**		**0.89**	**(0.85–0.94)**	**7.4 × 10^−6^**	**0.10**
**All combined**^d^	**10 519**	**107 876**		**0.91**	**(0.88–0.95)**	**1.0 × 10^−6^^e^**	**0.083**		**0.91**	**(0.88–0.95)**	**3.8 × 10^−7^^f^**	**0.091**

^a^Likelihood ratio test for heterogeneity.

^b^Estimated effective sample size 8237 cases and 8237 controls, after adjusting for genomic control.

^c^Estimated effective sample size 6441 cases and 6441 controls.

^d^Estimated effective sample size 14 678 cases and 14 678 controls, after adjusting for genomic control.

^e^*P*_adj_ conditioned on rs1035142 = 0.18.

^f^*P*_adj_ conditioned on rs700635 = 0.044.

In blood and sun-exposed skin tissues, rs700635[C] is associated with the presence of RNA isoforms of *CASP8* in which intron 8 fails to splice out correctly and contain a variant exon unique to the NM_033358 transcript. These RNA isoforms are of interest because they conceivably could lead to attenuated caspase-8 signalling (7). We analysed RNA-sequencing (RNA-seq) data from 93 samples in the Genotype-Tissue Expression (GTEx) project and found that rs700635[C] is similarly associated with a significant retention of *CASP8* sequences mapping to the 5′ half of intron 8 in both normal prostate and breast tissues (Fig. 1). A modest decrease in expression of the *CASP8* major exons was also shown in breast although not in prostate tissue. We also noted the presence of RNA-seq reads in the 3′ end of intron 7, as we had previously seen in sun-exposed skin (7). In order to determine whether similar splicing anomalies are present in tumours of the breast and prostate, we examined the cancer genome atlas (TCGA) RNA-seq data. Indeed, both tumour types showed highly significant retention of intron 8 sequences in association with rs700635[C] (*P* = 8.3 × 10^−80^ and 1.4 × 10^−34^ for breast and prostate tumours, respectively; Supplementary Material, Fig. S1).

**Figure 1.**
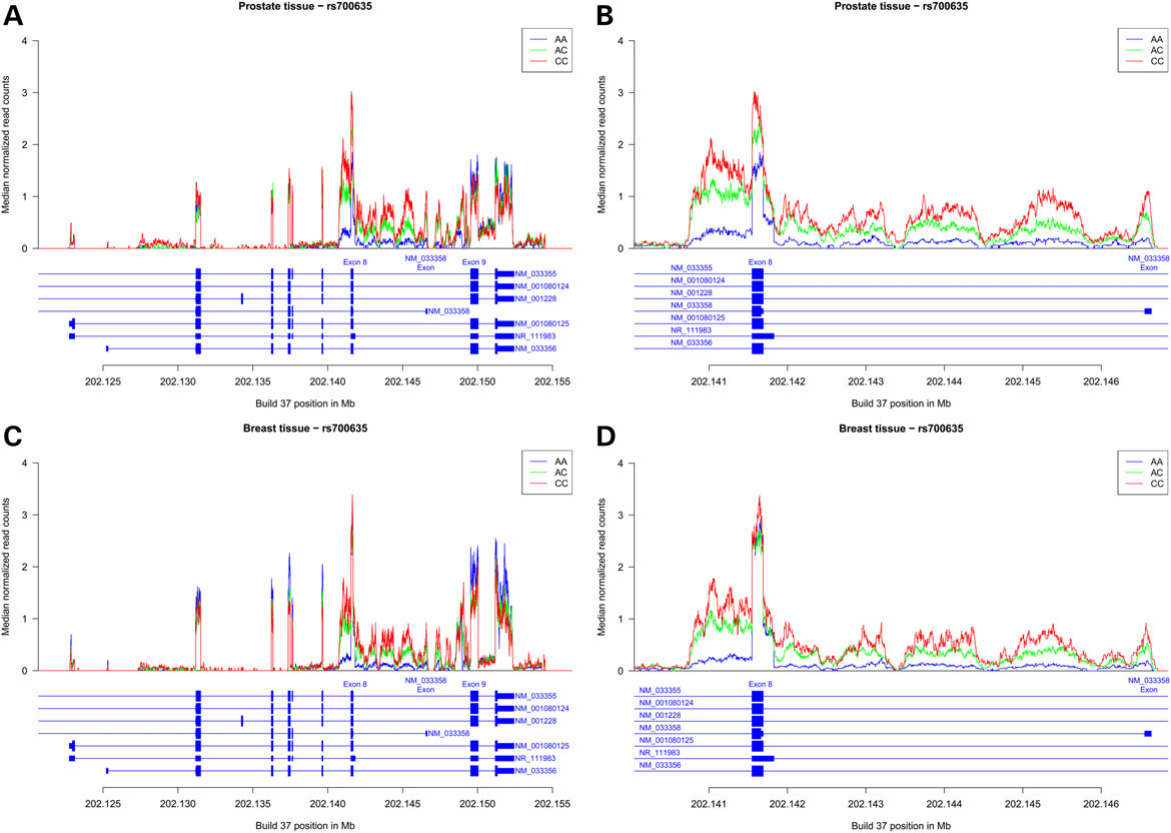
Carriers of the rs700635[C] allele show preferential retention of CASP8 intron 8 sequences. RNA-seq data were obtained from normal prostate or breast tissue of donors who were genotyped for rs700635. (**A**) Median normalized read counts (normalized for each individual to the total number of aligned reads) from prostate RNA were stratified by rs700635 genotype and plotted over the genomic region covering the coding exons of *CASP8*. Data are from *n* = 6 C/C homozygotes (shown in red), *n* = 12 A/C heterozygotes (shown in green) and *n* = 19 A/A homozygotes (shown in blue). The *X*-axis is the genomic position in Mb (GRCh37/hg19). The structure of RefSeq *CASP8* transcripts in the region is shown beneath the X-axis. (**B**) Zoom showing prostate RNA median normalized read counts by genotype over the region extending from the 3′ end of intron 7, through the 5′ region of intron 8 up to the NM_033358 variant exon. (**C**) Similarly plotted median normalized read counts from breast RNA. Data are from *n* = 7 C/C homozygotes, *n* = 27 A/C heterozygotes and *n* = 22 A/A homozygotes. (**D**) Zoom showing breast RNA median-normalized read counts over a region encompassing the 5′ region of *CASP8* intron 8. (**E**) Association between rs700635 genotype and prostate RNA-seq read count in intron 8. For each individual, the median of normalized RNA-seq read counts overlapping the 5′ region of intron 8 [chr2:202141827-202146555 (GRCh37/hg19)] was determined. Association with genotype was then determined by linear regression against rs700635 variant allele count. *P* = 3.8 × 10^−8^ and *β* = 0.33 (SD units). (**F**) Similarly determined association between rs700635 genotype and prostate RNA-seq read count over all major coding exons of *CASP8. P* = 0.678 and *β* = −0.07. (**G**) Association between rs700635 genotype and breast RNA-seq read count in the 5′ region of intron 8. *P* = 3.11 × 10^−11^ and *β* = 0.36. (**H**) Association between rs700635 genotype and breast RNA-seq read count over all *CASP8* major coding exons. *P* = 0.0475 and β = −0.30.

**Figure 1:**
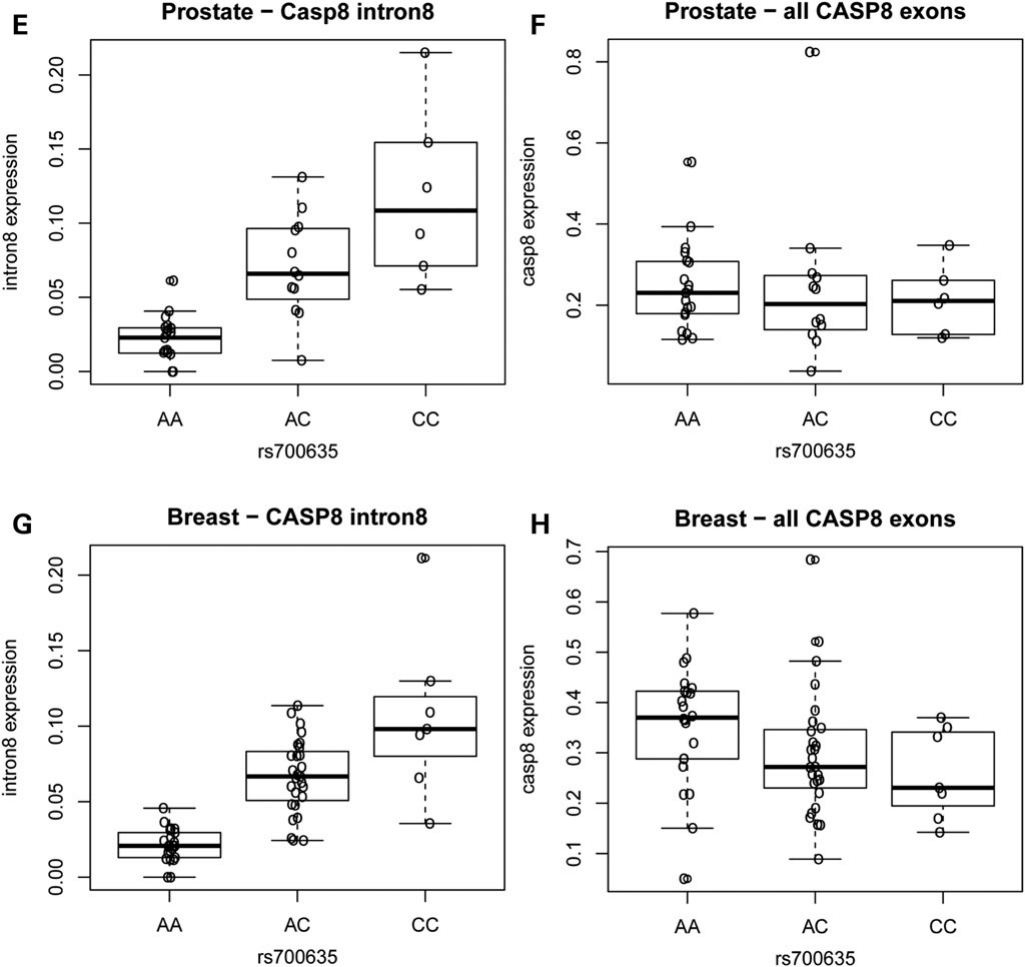
Continued

**Figure 2.**
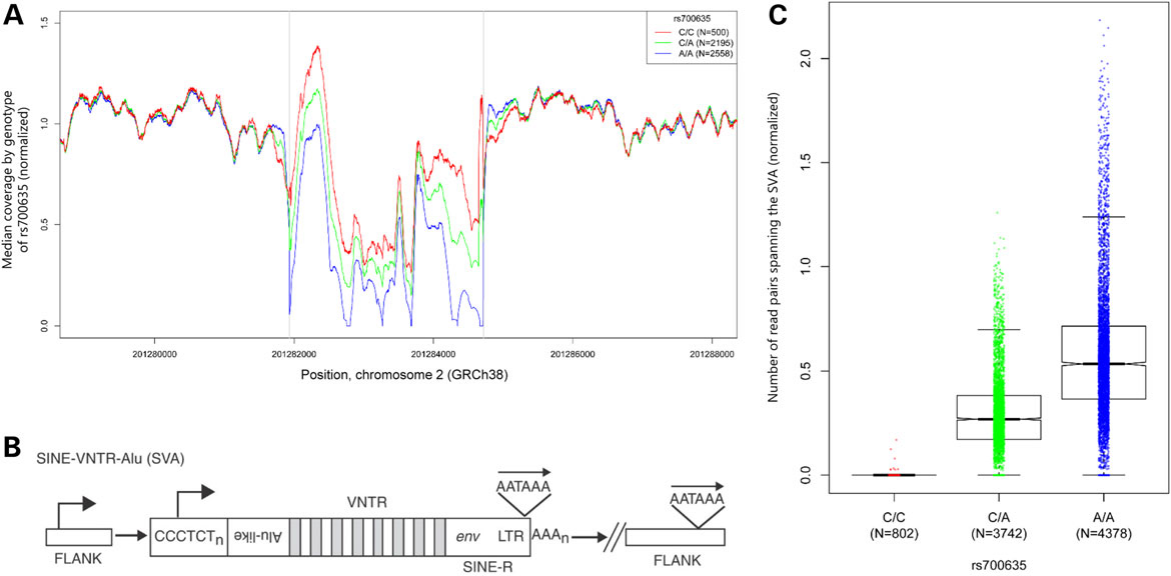
rs700635[C] is associated with insertion of an SVA-E retrotransposon into the CASP8 gene. (**A**) The DNA sequence coverage of the *CASP8* intron 8 region was normalized and the median coverage for each genotype was plotted at each position. A copy number variation was detected in the region chr2:201281936-201284718 (GRCh38/hg38). Linear regression of mean normalized coverage by SNP genotype showed a significantly higher copy number over this region in association with rs700635[C] (*P* << 2 × 10^−16^). (**B**) The general structure of an SVA element: these non-autonomous retrotransposons consist of a CCCTCT repeat region (which may have RNApolII promoter activity), followed by a series of *Alu*-like repeats, followed by a VNTR region, followed by a degenerate *env* gene fragment and long terminal repeat from an extinct endogenous human retrovirus (HERV-K). Transcription in the SVA may utilize a polyadenylation signal within the long terminal repeat, or it may continue into flanking chromosomal sequences (see text). [Image from (17), used with permission.] (**C**) Boxplot showing association of DNA-sequence read pairs spanning the SVA insertion site with rs700635 genotype: read pairs were selected such that the forward read was in *CASP8* sequence within an interval of 1000 bp to the left of the SVA-E insertion site and the paired reverse read was in *CASP8* sequence within an interval of 1000 bp to the right of the SVA-E insertion site. Such reads are expected to occur only if the SVA-E retrotransposon is absent. Counts of qualifying reads were normalized and plotted by genotype. Association was assessed by linear regression of read count *versus* rs700635 genotype (*P* << 2 × 10^−16^).

In both our RNA-seq data and our whole-genome DNA sequencing data, we observed very low read coverage over a ∼2.8 kb region at the 3′ end of intron 8. The region concerned is highly repetitive and GC rich. Using whole-genome sequence (WGS) data from 5253 individuals [from whom the majority of samples were prepared using Illumina's polymerase chain reaction (PCR)-free or TruSeq Nano sample preparation methods, which are less susceptible to GC bias than older HiSeq methods] we detected a copy number variation at this location, with significantly higher read counts being correlated with the presence of rs700635[C] (Fig. 2A). Further examination revealed that the region contains a polymorphic insertion/deletion of a 2792 bp short interspersed element-variable number of tandem repeat-*Alu* (SINE-VNTR-*Alu*), subfamily-E retrotransposon (SVA-E, Fig. 2B). The SVA-E retrotransposon, when present, is in the opposite orientation to the *CASP8* gene.

We used DNA sequence reads with paired ends near the SVA-E insertion site breakpoints to detect the presence of the retrotransposon and assess its association with rs700635 genotype. There was a strong association between reads that cross the (empty) insertion site and the rs700635[A] allele (Fig. 2C, *P* << 2 × 10^−16^). Similarly, reads that cross from native *CASP8* sequence into SVA-E retrotransposon sequence were strongly associated with the rs700635[C] allele (Supplementary Material, Fig. S2). Thus, rs700635[C] is associated with protection against prostate cancer, failure to correctly splice *CASP8* intron 8, and with the presence of the SVA-E retrotransposon in the intron.

**Figure 3.**
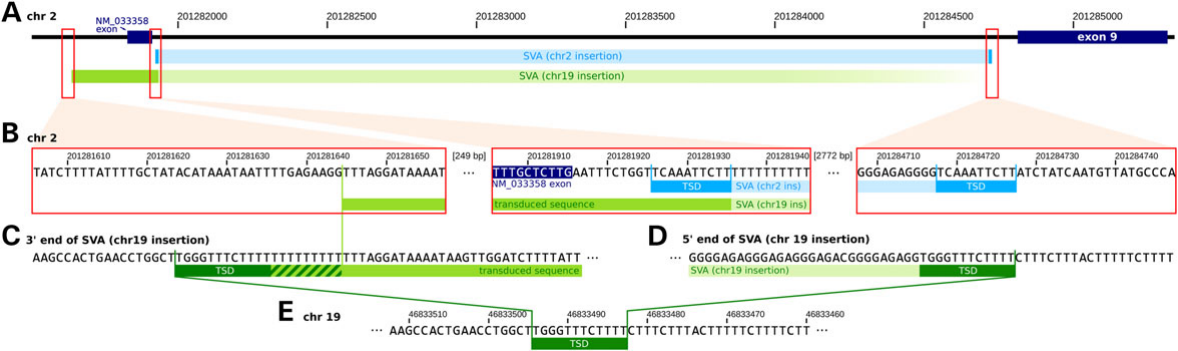
The SVA-E insertion site in the *CASP8* gene on chromosome 2 and the retrotransposition-transduction event on chromosome 19. Note that all structures are drawn to preserve the genomic orientation of the *CASP8* gene and the chr2 SVA-E insertion. Thus, the 5′ end of the SVA-E is to the right in the diagram and chr2 sequences are from the genomic forward strand. (**A**) The chr2 SVA-E (pale blue bars) is inserted into *CASP8* intron 8 between the NM_033358 variant exon and exon 9 (at chr2:201281936-201284718). A copy of the SVA-E is present on chr19 (green bars). The chr19 SVA-E is flanked by a transduced 288 bp segment, including the NM_033358 variant exon, from chr2 (darker green bar). (**B**) The structure of the chr2 breakpoints: the left panel shows the breakpoint between the transduced sequence and *CASP8* intron 8 sequence. The central panel shows the left end of the SVA-E insertion on chr2 (blue bars) with the TSD sequence indicated. The sequence of this junction is the same on the chr19 transduced copy (green bars). The right panel shows the breakpoint between the right end of the SVA-E and native *CASP8* intron 8 sequence with the TSD indicated. (**C**) The structure of the breakpoint between the transduced sequence and native chr19 sequence: the transduced *CASP8* intron 8 sequence contains a polyadenylation signal on the reverse strand (appearing here as TTTATT). Moving leftwards on the reverse strand, this is followed by a short polyA sequence (hatched green bar) then a TSD derived from native chr19 sequence. (**D**) The breakpoint between the 5′ end of the SVA-E and native chr19 sequence, with the TSD sequence indicated. (**E**) The structure of the chr19 insertion site when the SVA-E is not present. Note that here the TSD sequence is unduplicated.

We characterized the insertion site of the *CASP8* SVA-E on chromosome 2 (Fig. 3). The SVA-E is inserted 20 bp to the right of the NM_033358 variant exon and extends to within 112 bp of exon 9. The insertion is flanked by a 10 bp target site duplication (TSD). Incidentally, we noted some anomalies in the correlation between reads spanning the left and the right ends of the SVA-E insertion in certain individuals. Upon further investigation, it became evident that a copy of the SVA-E has transposed out of the *CASP8* gene and inserted into chromosome 19 (±6 bp around chr19:46833489, Fig. 3). It appears that when this transposition event occurred, the SVA-E transduced with it a 288 bp stretch of 3′ flanking sequence from the *CASP8* gene (which, due to the orientation of the SVA-E, is to the left of the insertion site, Fig. 3). SVA elements are known to occasionally transduce 3′ flanking sequences during transposition (10). For the *CASP8* element, the probable directionality of transposition was established by the observations that (i) the transduced segment exists as native chr2 sequence on copies of chromosome 2 which do not contain the SVA-E insertion, (ii) the transduced segment does not occur on chr19 except in association with the SVA-E insertion, (iii) the chr2 TSD comprises the first 10 bp of the transduced segment, (iv) on chr19, the transduced segment is followed by a short polyA tract then by a TSD comprised of native chr19 sequence and (v) the polyA tract is preceded in the transduced segment by an AATAAA polyadenylation sequence comprised of native *CASP8* intron 8 sequence. Taken together, these observations suggest that a transcript arising at the 5′ end of the chr2 SVA-E failed to terminate at the internal SVA-E polyadenylation site, but rather ran on into native chr2 sequence until it encountered a fortuitous polyA signal on the reverse stand of *CASP8* intron 8 sequence. The resulting RNA gained a short polyA tail then retrotransposed into the site on chr19, creating a new TSD at the insertion site. This indicates that the SVA-E insertion in the *CASP8* gene has been active and capable of retrotransposition. Ongoing activity of the SVA-E might interfere with the correct functioning of the *CASP8* gene.

We wanted to impute *CASP8* SVA-E insertion genotypes into our set of long-range phased Icelanders (11–13) and to genotype the SVA-E in non-Icelandic populations. Even though the association between the SVA-E retrotransposon and rs700635[C] is highly significant, the correlation is not perfect (we estimate the *r*^2^ as 0.71 in the Icelandic population). Genotyping the SVA-E directly is complicated by the chromosome 19 retrotransposition/transduction event described above. Nevertheless, by restricting genotype calls to information derived from insertion site-spanning and right end read pairs, we were able to determine SVA-E genotype likelihoods for a set of 8434 whole-genome sequenced individuals and impute these SVA-E genotypes for 150 656 chip-typed, long-range phased Icelanders and their relatives (see the ‘Materials and Methods’ section). We then searched for a SNP allele or haplotype that tags the presence of the SVA-E with high confidence. We identified rs1035142[T] (MAF = 0.38), which has an *r*^2^ of 0.999 with the SVA-E insertion in the Icelandic population. In order to validate this tagging SNP and see whether the linkage disequilibrium (LD) relation holds in non-Icelandic populations, we generated a Centaurus single-track assay for rs1035142 and a PCR assay to detect the SVA-E directly (see the ‘Materials and Methods’ section). The presence of the SVA-E showed perfect LD with rs1035142[T] in a set of 184 individuals of European ancestry from the USA (*r*^2^ = 1, *P* << 2 × 10^−16^). Accordingly, we concluded that rs1035142[T] is a reasonable surrogate for the SVA-E-positive (SVA-E[pos]) genotype in populations of European ancestry.

We then re-assessed the association with prostate cancer using knowledge of the SVA-E and its surrogate SNP. As expected, the SVA-E[pos] and rs1035142[T] genotypes gave similar association results for prostate cancer in the Icelandic population sample (*P* = 0.0069 and 0.0064, respectively). Inclusion of non-Icelandic replication sample genotypes yielded an overall result of OR = 0.91, *P* = 3.8 × 10^−7^ (cf. OR = 0.91, *P* = 1.0 × 10^−6^ for rs700635[C], Table 1). In conditional analysis, the association with rs1035142[T] remained nominally significant when adjusted for the effect of rs700635 (*P*_adj_ = 0.044).

Next, we examined the association of the SVA-E and its surrogate rs1035142[T] with the transcriptional and splicing anomalies that were observed in *CASP8* intron 8. Using WGS imputed genotypes and Icelandic RNA-seq data from 628 blood samples, we scanned the region surrounding rs700635 to identify variants that correlate most strongly with the retention of intron 8 sequences. The SVA-E and a cluster of correlated variants, including rs1035142, were the most significantly associated with intron retention (*P* = 2.2 × 10^−32^ for the SVA-E, *P* = 6.5 × 10^−32^ for rs1035142, Supplementary Material, Fig. S3A). In conditional analysis, the signal from rs700635[C] was fully explained by either SVA-E[pos] or rs1035142[T] whereas both SVA-E[pos] and rs1035142[T] retained significant associations when adjusted for rs700635[C] (Supplementary Material, Table S1). SVA-E[pos] and rs1035142[T] were also better able to explain the associated reduction in expression of the major *CASP8* isoforms observed previously (Supplementary Material, Table S1). However, when we carried out a similar analysis of blood RNA expression microarray data using a probe from the NM_033358 variant exon (14), the strongest expression quantitative trait locus mapped significantly better to rs700635 (Supplementary Material, Fig. S3B, Supplementary Material, Table S1). The reason for this discrepancy is not clear, but it suggests that genetic factors other than the SVA-E might affect expression of the NM_033358 variant exon as measured by the expression microarrays.

We investigated potential mechanisms by which the SVA-E might interfere with correct splicing of *CASP8* intron 8. SVA-E element insertions have been observed previously to create anomalous splice junctions (15). However, in *CASP8* RNA-seq data from blood, we did not detect any new splice sites associated with the presence of the SVA-E. No active splice donors or acceptors were detected within the body of the SVA-E.

We had previously shown that at least some of the RNA sequences from intron 8 have paired ends spliced from upstream *CASP8* exons, which establishes that their transcriptional orientation is on the sense strand (7). Now, we examined more directly the possibility that the SVA-E might generate antisense transcripts reading through the 5′ region of intron 8. We carried out RT-PCR on blood RNA using orientation-specific primers in intron 8 for first-strand synthesis, followed by digital droplet PCR. In samples where the SVA-E is present, sense transcripts through the 5′ region of intron 8 were over 20-fold more abundant than antisense transcripts (Supplementary Material, Fig. S4). Moreover, normalized sense transcription through the region was significantly related to the presence of the SVA-E (*P* = 8.8 × 10^−7^). We also detected substantial sense-strand transcription in the 3′ end of intron 7 (Supplementary Material, Fig. S4, see also Fig. 1), raising the possibility of cryptic promoter activation. However, intron 7 transcripts did not vary in association with SVA-E genotype in blood RNA (Supplementary Material, Fig. S4F). Nevertheless, we can conclude that intron 8 RNAs whose abundance is increased in association with the SVA-E insertion are in the sense orientation with respect to the *CASP8* gene. This is in agreement with our conclusions from the RNA-seq data.

## Discussion

A strength of our association studies based on WGS is that they give a reasonably complete view of all SNPs and small indels present in Iceland down to a frequency of ∼0.05% (8). Moreover, the sequence data make possible a detailed analysis of associated structural variants, as we have done here. Using these methods, we have found that the insertion of an SVA-E retrotransposon into the *CASP8* gene is associated with transcript anomalies and protects against prostate cancer. SVA retrotransposons are relatively young in evolutionary terms and the SVA-E family is found only in humans, where it is thought to be highly active (16). SVA insertions have been seen to cause gene disruption and consequent disease (17). For example, the insertion of an SVA-E into the 3′UTR of the *FKTN* gene disrupts the gene by creating an anomalous splice junction, leading to Fukuyama muscular dystrophy (15). SVA elements oriented on the sense strand of the genes into which they insert generally cause gene disruption by this mechanism, or by the insertion of new promoters or polyadenylation signals (17, 18). In *CASP8*, however, the SVA-E is inserted in the reverse orientation with respect to the gene and so it may have a less extreme effect. At least one case of an SVA element producing antisense RNA has been documented before. In the *SYNE2* gene, an intronic SVA-D appears to utilize an internal promoter to produce antisense transcripts that terminate at a polyadenylation signal in the adjacent intron (19). It is not known whether this *SYNE2* antisense RNA is pathogenic. Our study found that the *CASP*8 SVA-E does not produce substantial amounts of antisense RNA running through the 5′ region of intron 8. The transduction events we observed suggest that such antisense RNAs, if produced, are prone to terminate at the polyadenylation site in *CASP8* intron 8 non-coding strand sequence. If, as seems likely, the *CASP8* SVA-E causes the splicing and transcriptional anomalies that we observe, then mechanisms other than antisense transcription from the inserted element are probable.

Variants that confer risk of some cancers and protect against others have been observed before, for example in the *TERT-CLPTM1L* locus (20). Satisfactory mechanistic explanations have not been forthcoming. In addition to its apoptotic functions, *CASP8* has pleiotropic roles in necroptosis, inflammation, cell survival and motility (1,2,21). The effects of disturbance of *CASP8* pathways might, therefore, vary between different tissue and tumour types. The *CASP8* SVA-E retrotransposon described here is associated with increased risk of BCC and breast cancer, but decreased risk of prostate cancer. Yet the associated *CASP8* transcriptional anomalies are similar in all three corresponding normal tissues and in breast and prostate cancers. This raises provocative questions about the mechanism through which *CASP8* expression changes might be involved in the risk state for these three diseases.

## Materials and Methods

### Subjects

Approval for the Icelandic study was granted by the Icelandic National Bioethics Committee and the Icelandic Data Protection Authority. All participants provided informed consent. Affected individuals were identified through the ICR, which has maintained records of prostate cancer, breast cancer and melanoma diagnoses since 1955. Controls consisted of individuals selected from other ongoing association studies at deCODE who did not have a diagnosis of the cancer in question recorded in the ICR. In the Icelandic and non-Icelandic studies, it is not possible to rigorously exclude that some male controls might have had sub-clinical prostate cancer. Therefore, the effects we report could be somewhat underestimated.

The Dutch prostate study population consisted of two recruitment-sets of prostate cancer patients; Group A consists of 360 hospital-based patients recruited between January 1999 and June 2006 at the urology outpatient clinic of the Radboud university medical center (Radboudumc); Group B consists of 1085 cases recruited between June 2006 and December 2006 through a population-based cancer registry held by the Netherlands Comprehensive Cancer Organization. The average age at diagnosis for patients in Group A was 63 years (median, 63 years; range, 43–83 years). The average age at diagnosis for patients in Group B was 65 years (median, 66 years; range, 43–75 years). The control individuals were cancer free at recruitment and matched for age. They were recruited from within a population-based survey in which 9371 individuals participated from a total of 22 500 age and sex stratified, randomly selected inhabitants of Nijmegen initially contacted. All participants were of self-reported European descent. The study protocol was approved by the Institutional Review Board of Radboud University and all study subjects gave written informed consent. Samples were genotyped on Illumina HumanHap300, HumanHapCNV370, HumanHap610, 1 M or Omni-Quad bead chips at the deCODE genetics facility.

The Spanish study population consisted of prostate cancer cases recruited from the Oncology Department of Zaragoza Hospital in Zaragoza, Spain, from June 2005 to September 2008. All patients were of self-reported European descent. Clinical information including age at onset, grade and stage was obtained from medical records. The average age at diagnosis for the patients was 69 years (median, 70 years) and the range was from 44 to 83 years. The control individuals were recruited at the University Hospital in Zaragoza and were cancer free at the time of recruitment. Study protocols were approved by the Institutional Review Board of Zaragoza University Hospital. All subjects gave written informed consent.

In the UK, prostate cancer cases and controls were recruited within the ‘Prostate Testing for Cancer and Treatment’ trial (ProtecT) (22) and biological material collection was carried out within the ‘Prostate Cancer: Mechanisms of Progression and Treatment’ (ProMPT) study. Men aged 50–69 years were offered an appointment for counseling and prostate-specific antigen (PSA) testing. Recruitment took place at nine sites in the UK; 94 427 men agreed to participate in the study (50% of men contacted) and 8807 (∼9%) had a raised PSA level. Of those with raised PSA levels, 2022 (23%) were diagnosed with prostate cancer; 229 men (∼12%) had locally advanced (T3 or T4) or metastatic cancers, the rest having clinically localized (T1c or T2) disease. Men with a PSA level of ≥20 ng/ml were excluded from the trial. For the present study, 547 patients with PSA values >3 ng/ml and diagnosed with prostate cancer after undergoing a needle biopsy (average age at diagnosis is 63 years). Controls were 1889 men of average age 62 years with PSA values between 3 and 10 ng/ml, but not diagnosed with prostate cancer after undergoing a needle biopsy or with PSA values <3 ng/ml (and not biopsied). Ethical approvals for the ProtecT and ProMPT studies were obtained from Trent Multi-Centre Research Ethics Committee (MREC/01/4/025 and MREC/01/4/061). All subjects gave written informed consent prior to inclusion in the study.

The US prostate cancer cases were recruited from the Pathology Core of Northwestern University's Prostate Cancer Specialized Program of Research Excellence from May 2002 to May 2010. The average age at diagnosis for the patients was 60 years (median, 59 years) and the range was from 39 to 87 years. The controls were recruited as healthy control subjects for genetic studies at the University of Chicago and Northwestern University Medical School, Chicago. All patients and controls were of self-reported European descent. Study protocols were approved by the Institutional Review Boards of Northwestern University and the University of Chicago. All subjects gave written informed consent.

The Romanian study population consisted of prostate cancer cases recruited from the Urology Clinic ‘Theodor Burghele’ of The University of Medicine and Pharmacy ‘Carol Davila’ Bucharest, Romania, from May 2008 to November 2009. The average age at diagnosis for the cases was 70 years (median, 71 years) and the range was from 46 to 89 years. The controls were recruited at the General Surgery Clinic ‘St Mary’ and at the Urology Clinic ‘Theodor Burghele’ of The University of Medicine and Pharmacy ‘Carol Davila’ Bucharest, Romania. The average age for controls was 60 years (median, 62 years) with a range from 19 to 87 years. The controls were cancer free at the time of recruitment. All subjects were of self-reported European descent. Study protocols were approved by the National Ethical Board of the Romanian Medical Doctors Association in Romania. All subjects gave written informed consent.

### Genotyping and association analysis

Procedures for whole-genome sequencing, Illumina SNP chip genotyping, imputation and association testing have been described previously (7,8,23). In this study, we used WGS information from 8383 Icelanders to impute the genotypes of 150 656 Illumina chip-typed, long-range phased Icelanders and their ungenotyped relatives. Single-track genotyping was done using Centaurus assays (24). All genotyping was carried out at the deCODE genetics facility with standardized quality control and allele calling protocols. We estimated the effective sample size for each cohort as *N*_e_ cases and *N*_e_ controls with *N*_e_ = 2 × *N*_a_ × *N*_c_/(*N*_a_ + *N*_c_), which adjusts for the asymmetry in the number of cases, *N*_a_, and controls, *N*_c_. For Iceland we further adjusted *N*_e_ for the relatedness of cases and controls by dividing by the genomic inflation factor *λ*_g_ (=1.22 for prostate cancer).

### RNA-seq analysis

RNA sequencing reads for 628 Icelandic blood samples were aligned to *Homo sapiens* GRCh38/hg38 using TopHat (25) version 2.0.12 with a supplied set of known transcripts in GTF format (RefSeq hg38). TopHat was configured such that it attempts first to align reads to the provided transcriptome then, for reads that do not map fully to the transcriptome, it attempts to map them onto the genome. Read mapping statistics used for read count normalization were calculated using samtools version 1.1 (http://www.htslib.org). Mapped RNA-seq read data for 37 prostate and 56 breast tissues from the GTEx Release phs000424.v4.p1 from 17.01.2014 were downloaded from the GTEx consortium (26) (http://www.gtexportal.org/home/) via dbGAP. TCGA data from breast and prostate tumours were obtained from CGHub (27). We used 1056 breast cancer and 374 prostate cancer samples from the same numbers of participants, after filtering for those who had genotype data for rs6743068 (a surrogate marker of rs700635) from corresponding samples.

### Determination of association with DNA-seq coverage over the SVA insertion region

In order to generate a plot of the median coverage of DNA sequencing over the SVA-E insertion region, we counted the number of reads covering each position in the interval chr2:201230000–201290000 (GRCh38/hg38). We counted only reads with an average PHRED-scaled base calling quality of ≥20, those that were not marked as a duplicate, not clipped by ≥10 base pairs at both ends, and only the primary alignments. We computed the mean of local read counts over two intervals nearby the SVA-E insertion site chr2:201230000–201280000 and chr2:201285000–201290000 and used this mean for normalization within each individual. We then computed the median of the normalized read counts at every position in the interval chr2:201230000–201290000 for each set of individuals with the same SNP genotype. The resulting median normalized read counts are shown in the plot in Figure 2A. In order to assess the association between normalized read count and rs700635 genotype, the mean of the normalized coverage over the SVA-E insertion interval (chr2:201281936–201284718) was determined for each individual and then regressed against rs700635[C] allele count (with the sequencing platform included as a covariate). Analysis was based on 5253 whole-genome sequenced individuals taken from an earlier data freeze than the 8383 individuals on whom the disease-association testing was based.

### Determination of association between SNP genotype and DNA sequence read pairs that detect the presence or absence of the SVA-E retrotransposon

We used three strategies to assess the presence or absence of the *CASP8* SVA-E retrotransposon from whole-genome sequence data. Firstly, we considered read pairs that span the SVA-E insertion site, which should be observed only in the absence of the retrotransposon. Read pairs were counted if the forward read was in *CASP8* sequence within an interval of 1000 bp to the left of the SVA-E insertion site and the reverse read was in *CASP8* sequence within an interval of 1000 bp to the right of the SVA-E insertion site. Counts of qualifying reads were then normalized to the mean of local read counts as described above. The boxplot in Figure 2 shows these normalized read counts as a function of genotype. Secondly, we considered read pairs that cross from *CASP8* sequence into the left end of an inserted SVA-E element. In this case, read pairs were counted if the forward read was in *CASP8* sequence within an interval of 1000 bp to the left of the SVA-E insertion site and the reverse read was in the SVA-E element within 1000 bp of its left end. (Note here that we define the ‘left end’ of the SVA-E element as its 3′ end, because the retrotransposon is inserted in an inverted orientation relative to the *CASP8* gene.) Counts of qualifying reads were normalized and plotted by genotype as above. Thirdly, we considered read pairs that cross from the right end of an inserted SVA-E element into *CASP8* sequence. In this case, read pairs were counted if the forward read was in the SVA-E element and within 1000 bp of its right end and the reverse read was in *CASP8* sequences within an interval extending 1000 bp to the right of the insertion site. Counts of qualifying reads were normalized and plotted by genotype as above. We assessed the association between qualifying read count and genotype by linear regression of normalized qualifying reads against rs700635[C] allele count (with sequencing platform and insert size included as covariates).

The chromosome 19 transduction event was identified by reads crossing from the left end of the transduced chr2 sequence into native chr19 sequence (see Fig. 3). Subsequently, we identified a linked indel (at chr19: 46833095, GRCh38/hg38), which can be used reliably to detect the presence of the chr19 transduction event. We estimated a frequency of ∼0.213 for the chr19 transduction event in the Icelandic population. We also noted the presence of an SVA-E carrying transduced *CASP8* intron 8 sequence on an unmapped contig likely originating from chromosome 22 (*chr22_KI270733v1_random*). Due to its insertion into repetitive genomic DNA sequence, this transduction event could not be characterized further.

### Genotyping the SVA-E

We determined genotypes of the SVA-E using methods outlined in (28). Briefly, each individual may have one of three genotypes: SS, a homozygous carrier of the SVA-E element; SD, a heterozygous carrier of the SVA-E and the deletion or DD, a homozygous carrier of the deletion. For each individual *i*, the input to the genotyping consists of two counts of reads pairs, a count B*i* of read pairs that span from the SVA-E across the right breakpoint and a count C*i* of reads that span the (empty) insertion site. We compute the relative likelihood of the genotypes as:

$$L(Bi,Ci|SS)=E^{|Bi|}$$

$$L(Bi,Ci|SD)=1/2^{\left(|Bi|+|Ci|\right)}$$

$$L(Bi,Ci|DD)=E^{|Ci|}$$

Here, *E* is a constant, which we set to *E* = 0.001. Genotype likelihoods were determined for a set of 8434 whole-genome sequenced Icelanders. The genotypes were then imputed into a set of 150, 656 Icelanders using methods described previously (23).

### PCR assay for the SVA-E

Two assays were employed, one which detects the presence of the SVA-E and a second which detects the empty insertion site. The forward primer was the same for both assays: AATGGGGAGGGATAGAGAGG. This primer is located to the left of the 288 bp transduced region in *CASP8* intron 8. For the SVA-E-present assay, the reverse primer was CTGTGACCCTGCCAAATCC, located within the left end of the SVA-E element. For the SVA-E-absent assay, the reverse primer was TGTGGTCCATGAGTTGGTAGA, located in *CASP8* sequence to the right of the insertion site. PCR products were resolved on agarose gels and scored manually.

### Expression microarrays

Samples of total RNA from human peripheral blood (*N* = 1001) were hybridized to Agilent Technologies Human 25 K microarrays as described previously (14). We quantified expression changes between two samples as the mean logarithm (log_10_) expression ratio (MLR) compared with a reference pool RNA sample. In comparing expression levels between groups of individuals with different genotypes, we denoted the expression level for each genotype as 10^(average MLR)^, where the MLR is averaged over individuals with the particular genotype. We determined the significance by regressing the MLR values against the number of variant alleles carried. We took into account the effects of age, gender and differential cell-type count in blood as explanatory variables in the regression.

### Strand-specific quantitative RT-PCR

Total RNA was isolated from whole blood using Qiagen RNA Maxi kits and the concentration and quality of the RNA was determined with Agilent 2100 Bioanalyzers (Agilent Technologies). Orientation-specific first-strand cDNA was synthesized using the High Capacity cDNA Reverse Transcriptase kits (Life Technologies, Inc.). Primers were intron 8 forward: CCAGCAACTATTCTTATTCTACCATCA, intron 8 reverse: TGTAGAGCCTCTCTGGTTCAATTTTA, intron 7 forward: CAGGAGGCCCAGGTATTGG, intron 7 reverse: CACACAGGACCTCTCAGACTTCTG, each used in separate reactions (500 nM final concentration). Quantitation was then carried out by digital PCR using both the forward and reverse primers and fluorescent labelled probes for intron 8: 6FAM-ACAGGAAGTTTGTTTTCT-MGB and for intron 7: 6FAM-ACACTGACTTTACAACGATGG-MGB (Life Technologies) on a Bio-Rad QX200™ AutoDG™ Droplet Digital™ PCR System.

## Supplementary Material

Supplementary Material is available at *HMG* online.

## Authors’ Contributions

The study was designed and the results interpreted by S.N.S., B.K., J.G., F.Z, G.T. and K.S. Subject ascertainment and recruitment was carried out by S.N.S., J.G., G.V.E., B.A.A., K.R.B., K.K.H.A., I.M.vO., S.N., F.F., B.T.H., Q.H., J.L.D., F.C.H., P.D. I., I.E.C., I.N.M., V.J., I.O., T.J., J.G.J., L.T., R.B.B., T.R., U.T., D.M., D.E.N., W.J.C., J.I.M., L.A.K. and E.J. Sequencing, genotyping and expression analysis was done by S.N.S., J.G., A.J., A.S., H.J., A.M.K., G.M., O.T.M. and U.T. Statistical and bioinformatics analysis was done by S.N.S., B.K., J.G., F.Z., S.A.G., B.V.H., S.H.V., P.D.I, G.M., A.H., H.H., P.S., D.F.G., A.K. and G.T. The manuscript was drafted by S.N.S., B.K., J.G., F.Z. G.T. and K.S. All authors contributed to the final version of the paper. Principle collaborators for the case-control population samples were (Iceland), L.A.K. (Netherlands), J.I.M. (Spain), W.J.C. (USA), D.E.N. (UK) and I.N.M. (Romania).

## Funding

The UK arm of the study acknowledges support from the National Cancer Research Institute (NCRI) for the ProMPT study [National Institute of Health Research (NIHR) collaborative study grant G0500966/75466]. The NCRI is formed by the Department of Health, Medical Research Council and Cancer Research UK. The collaborations are supported by the University of Cambridge, Cancer Research UK and the NIHR-funded Cambridge Bio-medical Research Centre, Cambridge, UK. We also thank the NIHR, Hutchison Whampoa Limited, the Human Research Tissue Bank (Addenbrooke's Hospital) and Cancer Research UK. The Department of Health funded the ProtecT study through the NIHR Health Technology Assessment programme. We also acknowledge to support of the research staff in S4 who so carefully curated the samples and the follow-up data (Jo Burge, Marie Corcoran, Anne George and Sara Stearn). Professor Donovan, Professor Hamdy and Professor Neal are National Institute for Health Research (NIHR) Senior Investigators. The views and opinions expressed therein are those of the authors and do not necessarily reflect those of the Department of Health. The Austrian arm of the study acknowledges support from the Anniversary Fund of the Austrian National Bank (grant number 15079) and by the Medical and Scientific Fund of the Mayor of the City of Vienna (grant number 10077). We are grateful to the GTEx Consortium for early stage sharing of RNA-seq and genotypic data. The following acknowledgement refers to the GTEx data: The Genotype-Tissue Expression (GTEx) Project was supported by the Common Fund of the Office of the Director of the National Institutes of Health. Additional funds were provided by the NCI, NHGRI, NHLBI, NIDA, NIMH and NINDS. Donors were enrolled at Biospecimen Source Sites funded by NCI\SAIC-Frederick, Inc. (SAIC-F) subcontracts to the National Disease Research Interchange (10XS170), Roswell Park Cancer Institute (10XS171) and Science Care, Inc. (X10S172). The Laboratory, Data Analysis and Coordinating Center (LDACC) was funded through a contract (HHSN268201000029C) to The Broad Institute, Inc. Biorepository operations were funded through an SAIC-F subcontract to Van Andel Institute (10ST1035). Additional data repository and project management were provided by SAIC-F (HHSN261200800001E). The Brain Bank was supported by a supplements to University of Miami grants DA006227 and DA033684 and to contract N01MH000028. Statistical Methods development grants were made to the University of Geneva (MH090941 and MH101814), the University of Chicago (MH090951, MH090937, MH101820 and MH101825), the University of North Carolina – Chapel Hill (MH090936 and MH101819), Harvard University (MH090948), Stanford University (MH101782), Washington University St Louis (MH101810) and the University of Pennsylvania (MH101822). Funding to pay the Open Access publication charges for this article was provided by deCODE genetics/AMGEN.

## Supplementary Material

Supplementary Data
