# Supplementary material for: Insertion of an SVA-E retrotransposon into the CASP8 gene is associated with protection against prostate cancer
Source: Hum Mol Genet. 2016 Jan 5;25(5):1008–18. doi: 10.1093/hmg/ddv622 (PMC4754045; doi:10.1093/hmg/ddv622)
Supplement: Supplementary Data [file supp_25_5_1008__index.html]

Insertion of an SVA-E retrotransposon into the CASP8 gene is associated with protection against prostate cancer — Insertion of an SVA-E retrotransposon into the CASP8 gene is associated with protection against prostate cancer — Supplementary Data 

# Insertion of an SVA-E retrotransposon into the *CASP8* gene is associated with protection against prostate cancer

## Supplementary Data

Supplementary Data

- Supplementary Data - Docx file
