# Supplementary material for: Insertion of an SVA-E retrotransposon into the CASP8 gene is associated with protection against prostate cancer
Source: Hum Mol Genet. 2016 Jan 5;25(5):1008–18. doi: 10.1093/hmg/ddv622 (PMC4754045; doi:10.1093/hmg/ddv622)
Supplement: Supplementary Data [file supp_ddv622_ddv622supp.docx]

**Supplementary Material for Simon N. Stacey et al: Insertion of an SVA-E retrotransposon into the *CASP8* gene is associated with protection against prostate cancer.**

**Supplementary Table 1: Conditional analysis of CASP8 RNA expression associations with genotype.**

|  | **SVA-E** | | | **rs1035142** | | | **rs700635** | | |
| --- | --- | --- | --- | --- | --- | --- | --- | --- | --- |
| **RNA expression type** | **r^2^** | ***P*** | ***P_adj_^a^*** | **r^2^** | ***P*** | ***P_adj_^a^*** | **r^2^** | ***P*** | ***P_adj_^b^*** |
| CASP8 intron 8 retention (RNAseq)^c^ | 0.179 | 4.9x10^-18^ | 5.7x10^-6^ | 0.180 | 3.8x10^-18^ | 4.7x10^-6^ | 0.134 | 2.2x10^-14^ | 0.38 |
| CASP8 major exon expression (RNAseq) | (-)0.067^d^ | 2.7x10^-7^ | 3.5x10^-4^ | (-)0.180^d^ | 2.1x10^-7^ | 2.6x10^-4^ | (-)0.035^d^ | 1.8x10^-4^ | 0.48 |
| NM_033358 variant exon expression (Microarray) | 0.336 | 3.4x10^-35^ | 0.011 | 0.343 | 6.7x10^-36^ | 0.0069 | 0.420 | 5.1x10^-46^ | 2.6x10^-13^ |
| Conditional analyses were performed on 370 blood RNA samples that had been assayed on both RNAseq and expression microarray platforms. Genotypes were determined by WGS and imputation. | | | | | | | | | |
| ^a^*P* adjusted for the effect of rs700635. |  |  |  |  |  |  |  |  |  |
| ^b^*P* adjusted for the effect of rs1035142. |  |  |  |  |  |  |  |  |  |
| ^c^quantified over the 5´ region of intron 8 (chr2:201277104-201281832 [GRCh38/hg38]). | | |  |  |  |  |  |  |  |
| ^d^(-) indicates that the direction of effect is opposite to the other two RNA expression types. | | |  |  |  |  |  |  |  |

**Supplementary Figures:**

**Supplementary Figure 1:**


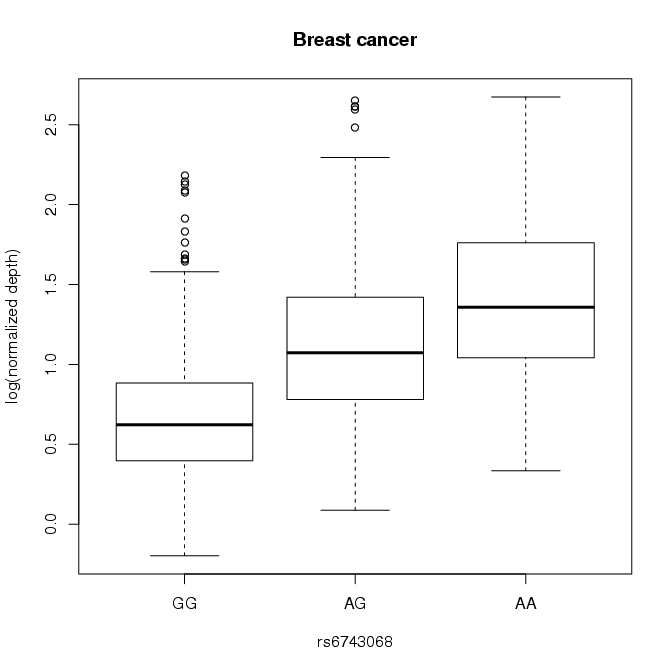

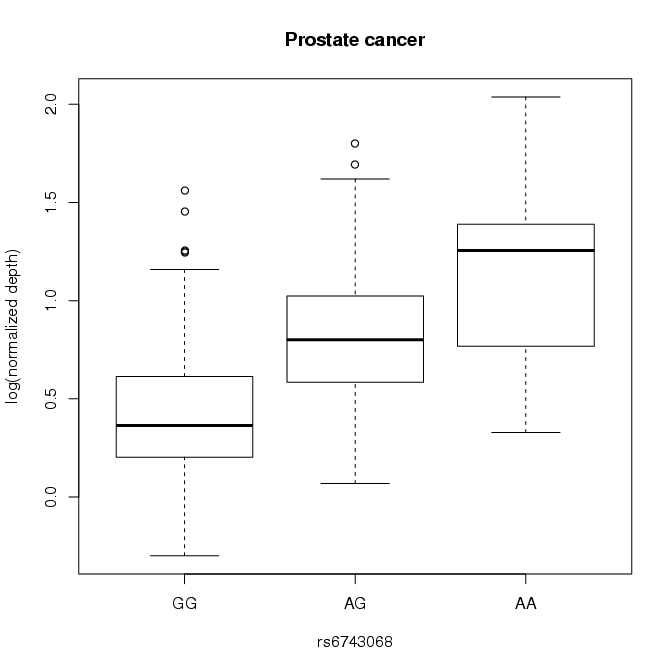


a

b

**Supplementary Figure 1 Legend:** **Association of rs700635 surrogate rs6743068 with CASP8 intron 8 sequence retention in prostate and breast tumours.** For each individual tumour, the median of normalized RNA-seq read counts overlapping the 5´ region of intron 8 (chr2:202141827-202146555 [hg19/Build 37]) was determined. Association with genotype was then assessed by linear regression against rs6743068 variant allele count. rs6743068 was selected because it is in strong linkage disequilibrium with rs700635 (r^2^=1 in Iceland) and it is present on the Affymetrix chips used for TCGA genotyping. (**a**) association for breast tumours based on n= 537 G/G homozygotes, n= 406 A/G heterozygotes and n= 102 A/A homozygotes. *P* = 8.3 x 10^-80^, β = 0.42. (**b**) association for prostate tumours based on n= 208 G/G homozygotes, n= 143 A/G heterozygotes and n= 23 A/A homozygotes. *P* = 1.4 x 10^-34^, β = 0.38.

**Supplementary Figure 2:**


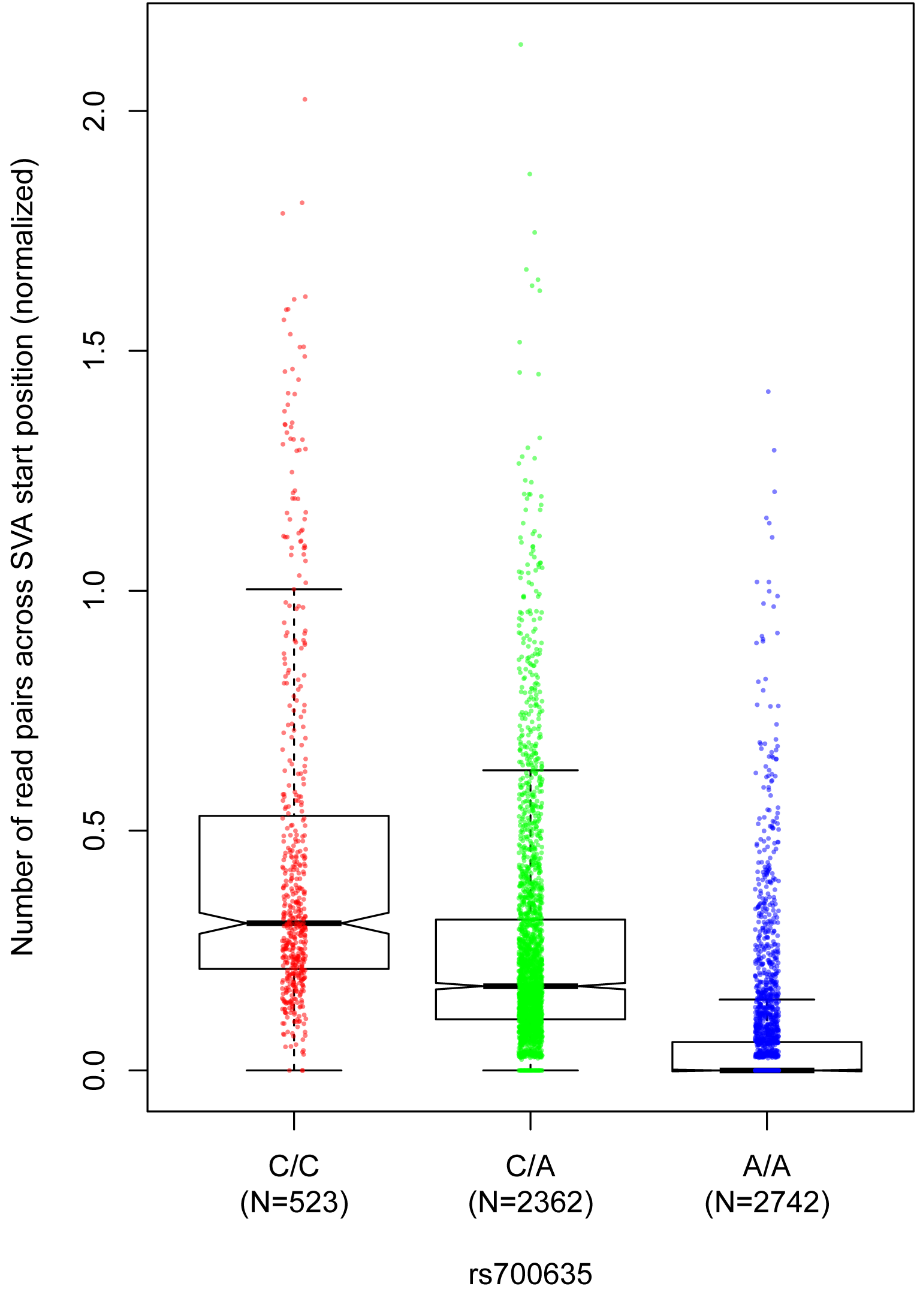

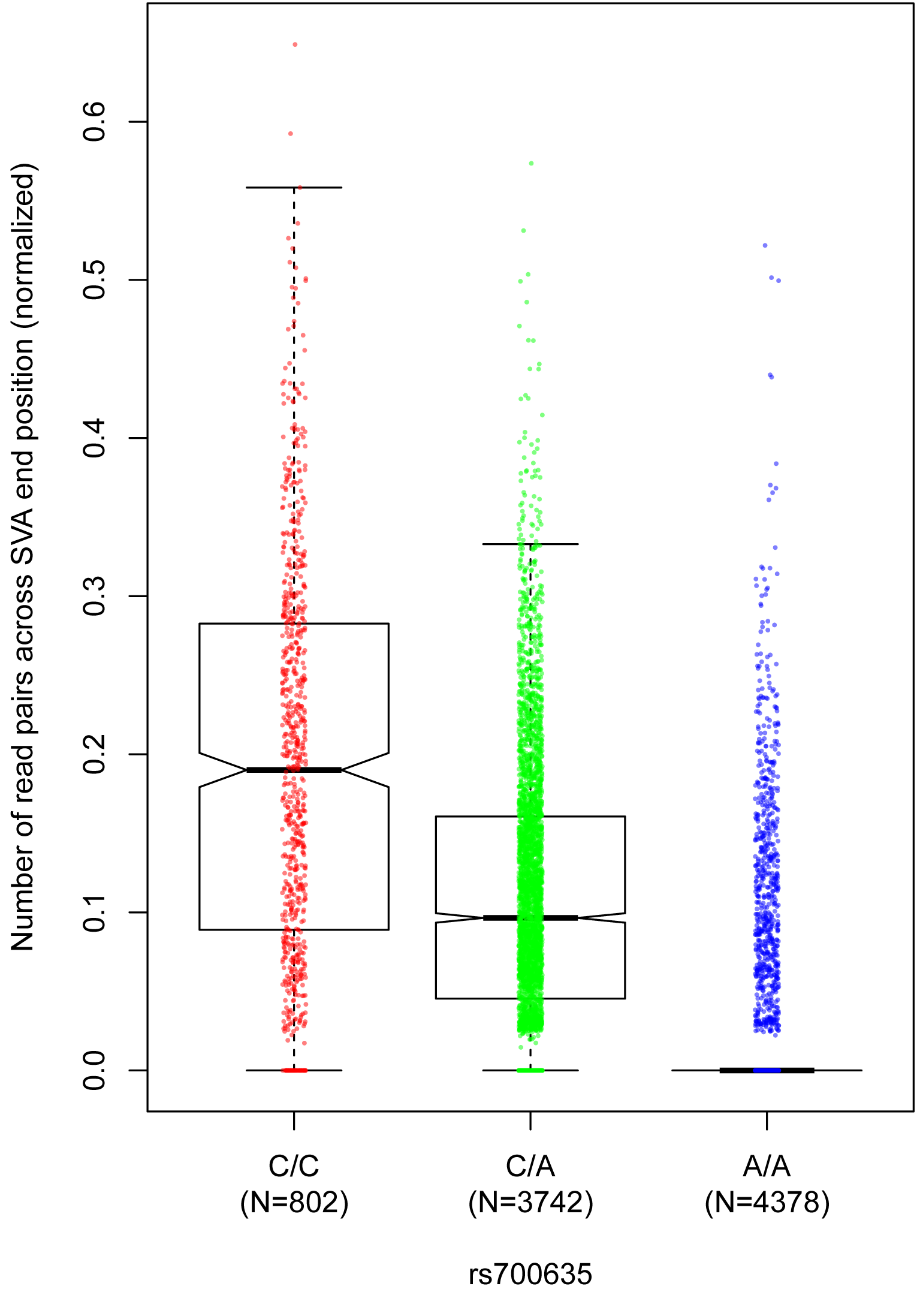


**a**

**b**

**Supplementary Figure 2 Legend:** **rs700635[C] is associated with insertion of an SVA-E retrotransposon into the *CASP8* gene.** (**a**) Boxplot showing association with rs700635 genotype of DNA sequence read pairs that cross from *CASP8* sequence into the left end of an inserted SVA-E element. Read pairs were selected if the forward read was in *CASP8* sequence to the left of the SVA-E insertion site and the paired reverse read was within the SVA-E element. Such reads are expected to occur only if the SVA-E retrotransposon is present. Counts of qualifying reads were normalized and plotted by genotype. Association was assessed by linear regression of read count *vs* rs700635 genotype (P << 2 x 10^-16^). Note that individuals showing evidence of the chromosome 19 transduction event (see text) were omitted from this analysis. (**b**) Boxplot showing association with rs700635 genotype of DNA sequence read pairs that cross from the right end of an inserted SVA-E element into *CASP8* sequence. Read pairs were selected if the forward read was in the SVA-E element and the paired reverse read was in *CASP8* sequence to the right of the SVA-E insertion site. Reads were normalized and regressed on genotype as above (P << 2 x 10^-16^).

**Supplementary Figure 3:**


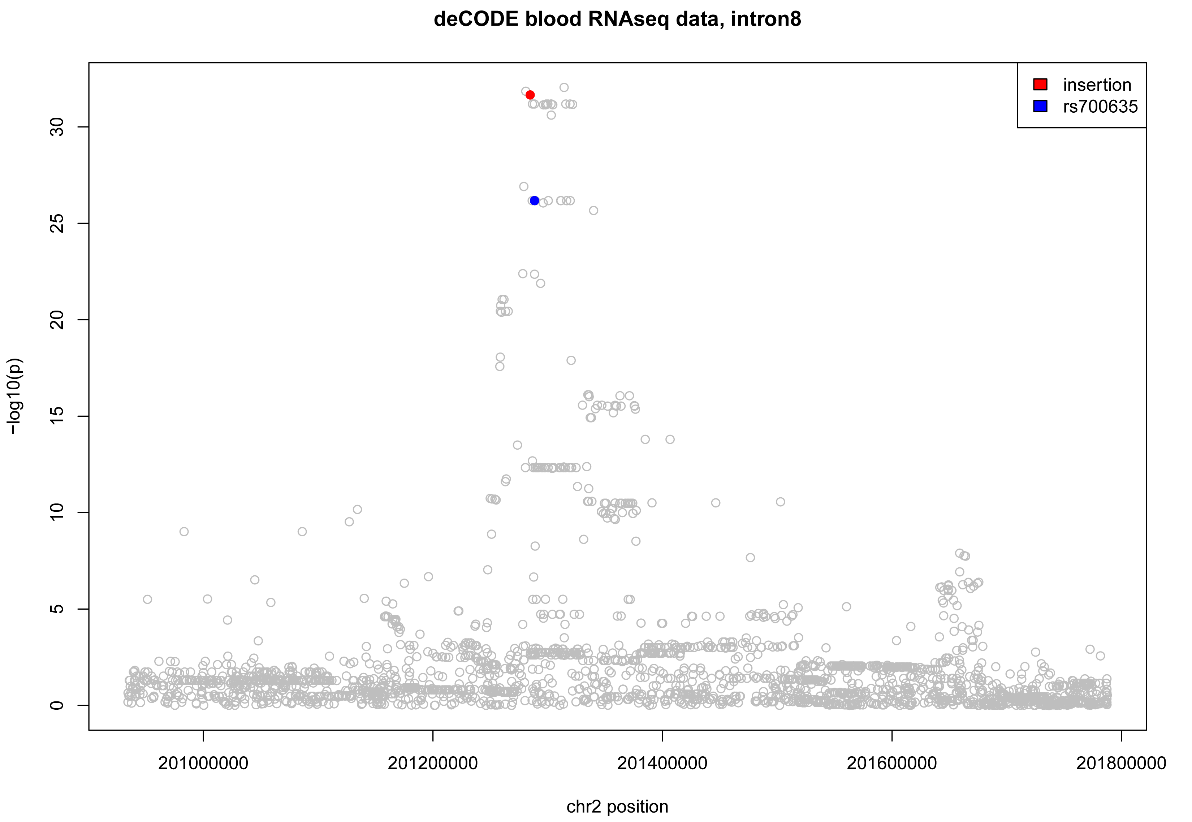

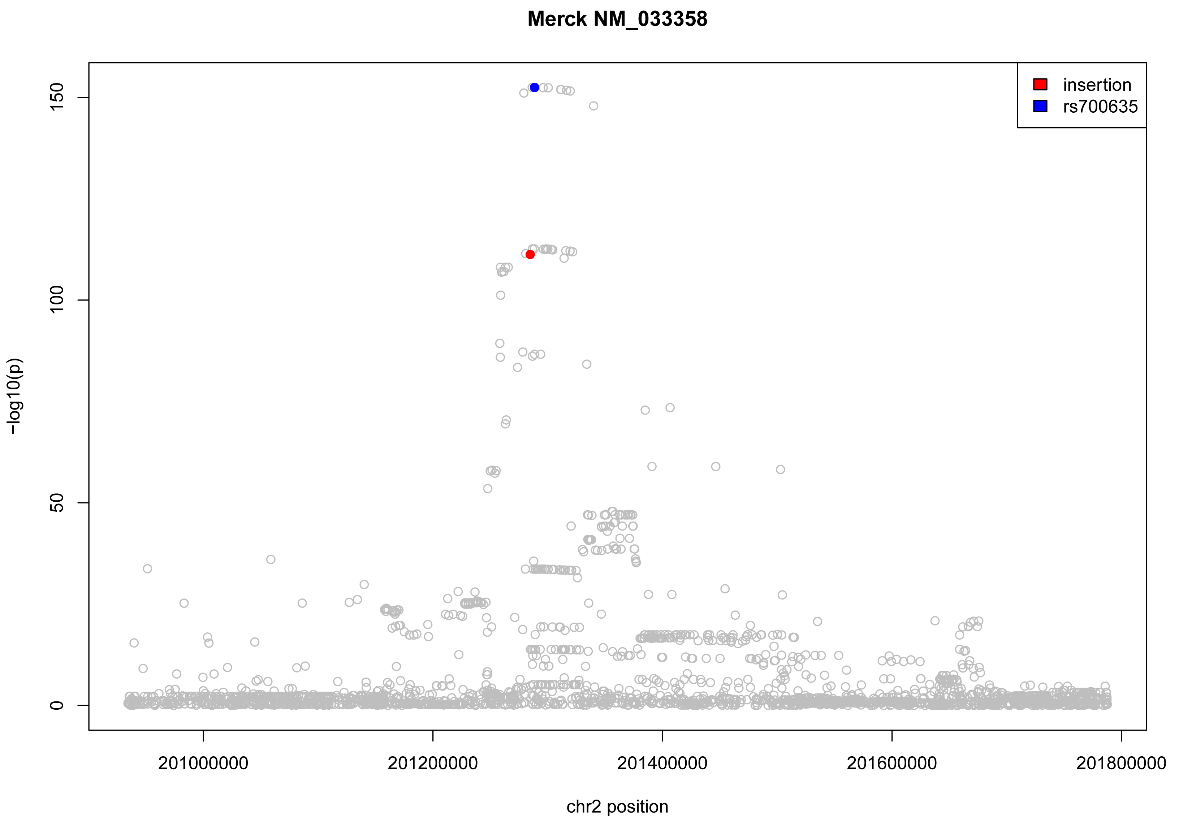


**b**

**a**

RNAseq data, CASP8 intron 8 retention

Microarray data, NM_033358 exon expression

**Supplementary Figure 3 Legend: Association of expression of *CASP8* intron 8 sequence with regional variants detected by whole-genome sequencing and imputation.** (**a**) shows association between blood-derived RNA-seq read count in intron 8 and regional variants. For each individual (n = 628), the median of normalized RNA-seq read counts overlapping the 5´ region of intron 8 (chr2:201277104-201281832 [GRCh38/hg38]) was determined. The association with genotype of each variant was then determined by linear regression of median normalized RNAseq read count against variant allele count. Association *P*-values were then plotted (as –log_10_ *P*) with the position on the X-axis corresponding to the location of the variant tested. (**b**) shows association between blood-derived RNA microarray expression levels from n = 1001 individuals, measured by a probe in the NM_033358 variant exon and regional variants. Mean log expression ratios were regressed against variant allele count for each variant tested and *P* values plotted (as –log_10_ *P*) against the position of the variant tested.

**Supplementary Figure 4:**

a

b

c

d


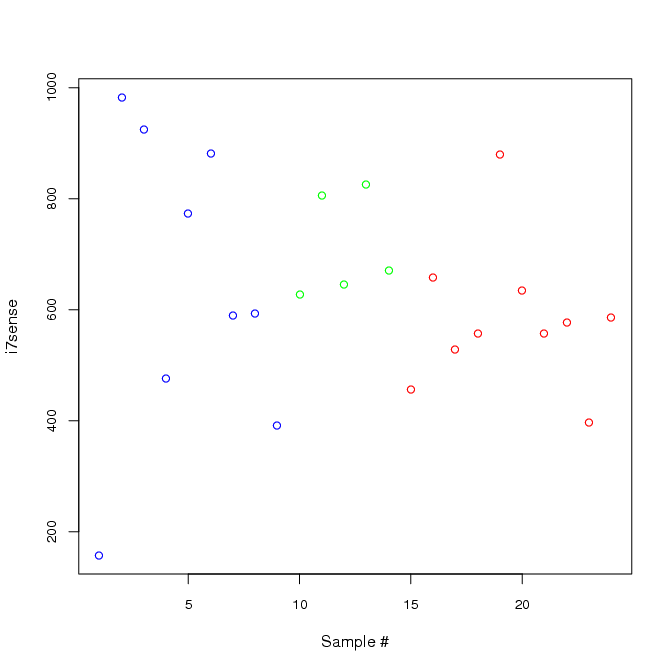

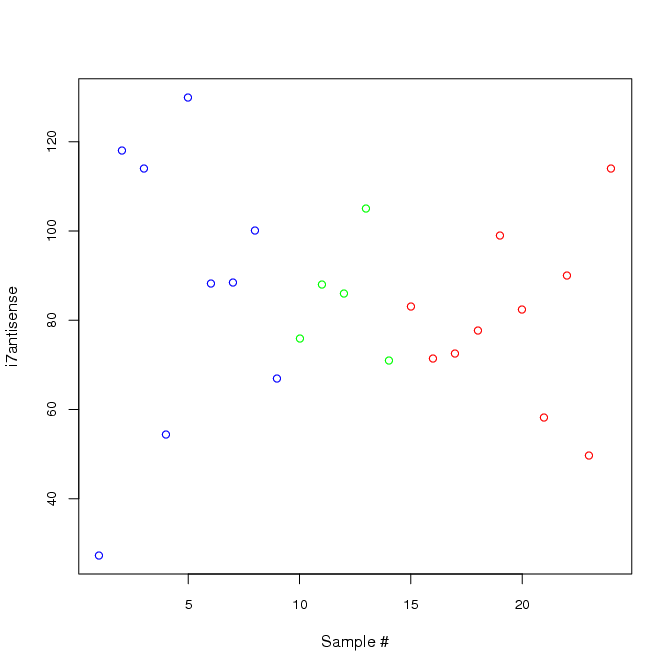

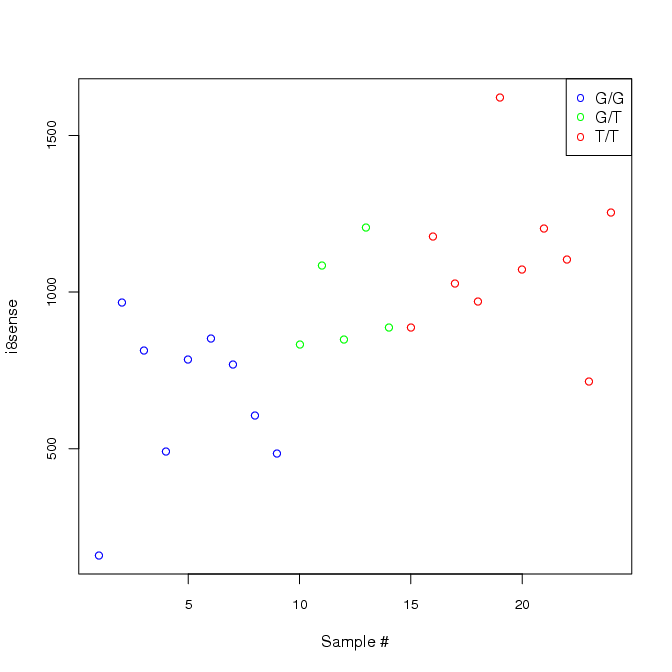

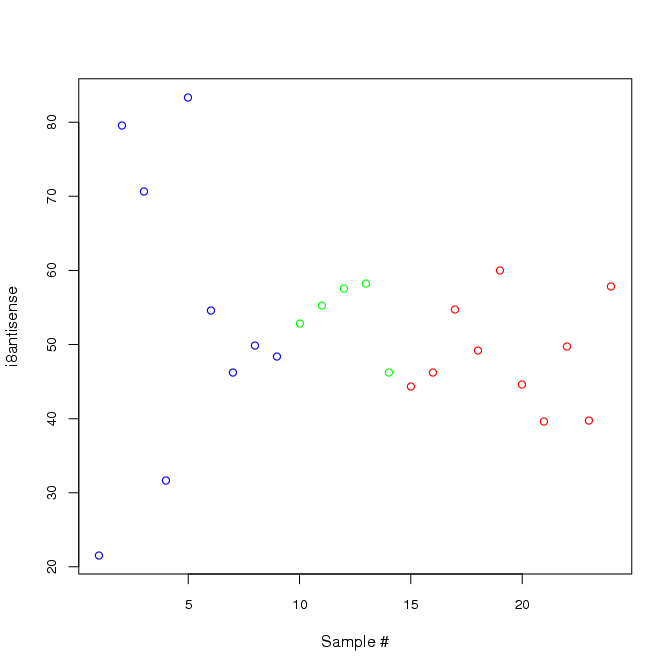

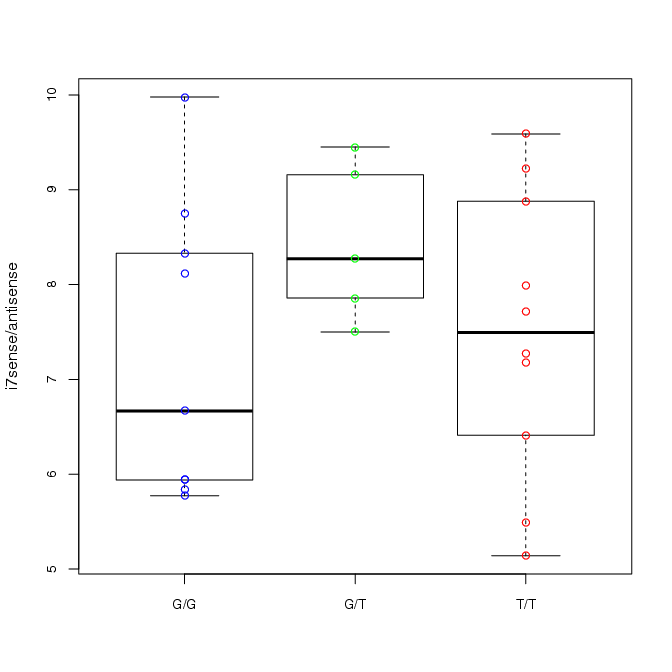


e

f


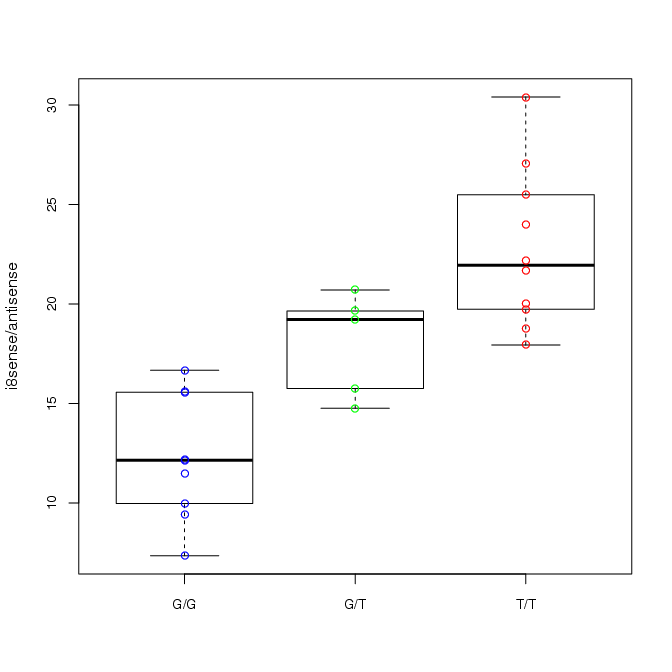


**Supplementary Figure 4 Legend: Strand-specific transcription in the *CASP8* intron 8 and intron 7 regions in relation to SVA-E insertion genotype.** Droplet RT-PCR was carried out using strand-specific primers to detect sense and antisense transcription in 24 samples of blood RNA from individuals with known genotype for rs1035142. The [T] allele corresponds to SVA-E[pos]. (**a**) Abundance of sense-strand transcripts in the *CASP8* intron 8 (5´portion, covering chr2: 201279218-201279296 [GRCh38/hg38]). (**b**) Abundance of antisense transcripts in the same segment of *CASP8* intron 8. Paired-sample *t*-test for sense *vs* antisense intron 8 transcript abundance *P* = 7.9 x 10^-13^ (**c**) Abundance of sense-strand transcripts in the 3´end of *CASP8* intron 7 (covering chr2:201276608-201276674). (**d**) Abundance of antisense transcripts in the 3´ end of *CASP8* intron 7. Paired-sample *t*-test for sense *vs* antisense intron 7 transcript abundance *P* = 1.3 x 10^-13^ (**e**) Expression of sense transcripts in the 5´portion of intron 8 normalized against antisense transcripts in the same region. Association with rs1035142 genotype was assessed by linear regression. *P* = 8.8 x 10^-7^. (**f**) Expression of sense transcripts in the 3´end of intron 7 normalized against antisense transcripts in the same region. Association with rs1035142 genotype was assessed by linear regression. *P* = 0.762.
